# Dietary intake and diet quality by weight category among a racially diverse sample of women in Birmingham, Alabama, USA

**DOI:** 10.1017/jns.2020.51

**Published:** 2020-12-07

**Authors:** Rebecca B. Little, Renee Desmond, Tiffany L. Carson

**Affiliations:** 1 Division of Preventive Medicine, Department of Medicine, School of Medicine, University of Alabama at Birmingham, Birmingham, AL, USA; 2 O'Neal Comprehensive Cancer Center, University of Alabama at Birmingham, Birmingham, AL, USA

**Keywords:** Dietary intake, Diet quality, Women, Healthy Eating Index, Deep South

## Abstract

Diet is a modifiable contributor to health. The lack of adherence to recommended dietary guidelines may contribute to the disproportionate burden of obesity and other chronic conditions observed in the Deep South region of the United States. The objective of this cross-sectional study was to describe food group intake and diet quality by race and weight status of women in the Deep South. Study participants were eighty-nine healthy female volunteers (56 % black, 44 % white, mean age 39⋅7 ± 1⋅4 years) recruited from Birmingham, AL, USA. Body Mass Index (BMI) determined weight status (non-obese/obese). Healthy Eating Index-2010 (HEI-2010) calculated from dietary recalls assessed diet quality. Wilcoxon sum-rank test compared HEI-2010 scores by race and weight status. *χ*^2^ analysis compared the percentage of women who achieved maximum points for HEI-2010 index food components by subgroup. Caloric and macronutrient intake did not differ by race or weight status (mean kcal 1863⋅0 ± 62⋅0). Median Total HEI-2010 Score for the sample was 51⋅9 (IQR: 39⋅1–63⋅4). Although there was no statistical difference in diet quality by race, more whites achieved the maximum score for vegetable intake compared to blacks, while blacks reported higher total fruit intake. Non-obese women reported better diet quality (56⋅9 *v.* 46⋅1; *P* = 0⋅04) and eating more whole fruits, and more achieved the maximum score for protein from plant and seafood sources. In summary, differences in diet quality were observed by weight status, but not race among this sample. These results point to tailored dietary interventions for women in metropolitan areas of Alabama, USA.

## Introduction

The USDA Dietary Guidelines for Americans (DGA) are based on previous nutrition studies and aim to reduce diet-related chronic disease^(1)^. Unfortunately, adherence is less than optimal nationwide^(2)^ and the same holds true for the Southern region of the United States (US)^(3–6)^. This lack of adherence to recommended dietary guidelines may contribute to the disproportionate burden of obesity^(7)^ and other chronic conditions observed in the Deep South^(8,9)^. Within this regional disparity, there are also racial differences in weight and disease status that may be partially attributable to dietary intake. Previous research has shown that black women in the Deep South do not adhere to dietary guidelines^(10)^ and that, nationally, food group intake differs by race^(11)^. In the South, the literature yields mixed results for racial differences in diet quality^(3–5)^. Furthermore, half of these studies are limited to late adulthood^(3,6)^ and only one report compares the diet quality of women by race^(4)^. Health disparities when comparing black and white women are well-documented (i.e. obesity^(12)^, diabetes^(8)^ and colorectal cancer^(9)^), especially in the South^(8,9)^, and differences in diet quality offer one potential explanation for these observed disparities.

Diet quality is commonly quantified with diet indices by evaluating the complete diet of individuals or groups against the conformance to dietary guidelines, which also allows for the comparison between studies^(13)^. The Healthy Eating Index (HEI) is a frequently used diet index: the original edition boasts biochemical validation^(14)^ and revised versions are periodically released to reflect the most recent DGA^(15–17)^. These recommendations promote adequate intake of whole grains, fruits, vegetables and protein through a variety of sources (i.e. plants and seafood), and moderation of empty calories, sodium and refined grains, while allowing flexibility in eating patterns (i.e. individual preferences, cultural and ethnic influences, and vegetarianism)^(1,15)^.

Obese individuals are more likely to consume a lower HEI scoring dietary intake^(18)^. However, the relationship between diet quality and health remains complicated. Better diet quality is linked to reduced risk of cardiovascular disease^(19)^, obesity^(20,21)^, some cancers^(13,22)^ and all-cause mortality^(23)^. Even in the presence of obesity, individuals with better diet quality are less likely to have a chronic disease^(20)^, signifying comorbidities of obesity may be modulated by diet. Reversely, poor diet quality is a contributing factor to some chronic diseases in the absence of excess weight^(1)^.

One of the earliest studies comparing HEI by race found more white than black adults achieved an ideal HEI total score^(18)^. Later, Hiza *et al.*^(11)^ found mean HEI total score did not differ between white and black adults, although component scores differed by race. Notably, white adults consumed more vegetables, whole grains and dairy, while black adults achieved higher scores for moderation (i.e. lower saturated fat and sodium intake)^(11)^. In the South, studies differ on if the HEI scores are higher for black^(3)^ or white adults^(5)^, albeit the diets of both need improvement. Previously, a better diet assessed by HEI was associated with a decreased risk of cardiovascular and other disease-related death in both black and white men and women in the South^(4)^. Given the higher prevalence of obesity^(7)^ and nutrition-related diseases^(8,9)^ in the South, more research is needed to quantify the diet quality in this region.

To our knowledge, the overall diet quality of women of all ages in the Birmingham area has not been quantified, and comparisons of the diet quality between black women and white women in the Deep South are limited. Therefore, the purpose of this study was to assess diet quality with HEI-2010 and compare HEI-2010 scores of women by race and weight status. Given the health disparities by race in the Deep South and weight status, along with their diet interactions, we expected to find better diet quality in white than black women and non-obese than obese women. Findings from this research may inform public health promotion and practice in Alabama, USA.

## Materials and methods

### Study design and setting

This study is an ancillary analysis of data derived from a cross-sectional study of generally healthy female volunteers from the seven counties of the Birmingham, Alabama Metropolitan Statistical Area. For the parent study, participants provided demographic, anthropometric, survey and dietary data to examine associations with the gut microbiome using collected faecal samples. Methods and primary outcomes of this study are reported elsewhere^(24)^. For this analysis, dietary intake and diet quality are the primary outcomes.

This study was conducted according to the guidelines laid down in the Declaration of Helsinki and all procedures involving human subjects were approved by the UAB Institutional Review Board (FWA00005960). Written informed consent was obtained from all subjects.

### Participants

A total of 106 women participated in the parent study between March 2014 and August 2014. Inclusion criteria included being either a non-Hispanic black or non-Hispanic white woman aged 19 years or older with no major illness (e.g. cancer). A woman was excluded if she was pregnant, a current smoker, or unable to read or write. After additional exclusion for diet concerns described under Diet Assessment, the sample size for this study was *n* 89.

### Data collection and definitions

Self-reported demographic data included race, age, income (<$10 000, $10 000–$19 999, $20 000–$29 999, $30 000–$39 999, $40 000–$49 999 or ≥ $50 000), education (≤high school/GED, college or postgraduate) and marital status (single, married, separated, divorced or widowed). Race was self-identified as non-Hispanic black or non-Hispanic white. Demographics and anthropometrics were assessed at the first visit and dietary assessment was collected approximately a week later at the second visit.

### Anthropometrics

Trained personnel measured participants’ weight and height using a standardised protocol. Weight and height were measured using a calibrated 2-in-1 measuring station (Seca 284 measuring station, Hanover, MD) in light-weight clothing without shoes. Body Mass Index (BMI) was calculated as weight (kg)/height (m^2^).

### Diet assessment

The National Cancer Institute (NCI) Automated Self-Administered 24-Hour Dietary Assessment Tool (ASA24) version 2011 was used to collect one 24-h diet recall in person with the assistance of a trained data collector. ASA24 is administered as a multiple-pass standardised interview and provides a series of prompts with multi-level food probes to assess food types and amounts. The programme computes total intake, macronutrient composition, nutrient and food group estimates. Because daily dietary intake fluctuates, recalls are often not excluded for extreme caloric intake. However, with a single 24-h recall, a more conservative approach was used to avoid days of more extreme intake. Participants who did not report calories within 600–4400 kcal were classified as outliers by NHANES data^(25)^ and were excluded from the current analysis (*n* 3). Second, NHANES guidelines for portion and nutrient outliers (grams of protein and fat, Vitamin C, and Beta-carotene) were used to triaged records for further review. An additional *n* 14 participants were excluded from the analysis due to concerns for diet record validity (e.g. type or amount of food reported).

### Healthy Eating Index-2010 (HEI-2010)

For this study, the HEI-2010 scoring system was used to assess diet quality. HEI-2010 is an *a priori* scoring system based on the 2010 DGA, the most current recommendations in place during the conduct of the study.

The HEI-2010 separates food intake into twelve components: nine adequacy components (Total Fruit, Whole Fruit, Total Vegetables, Greens and Beans, Whole Grains, Dairy, Total Protein Foods, Seafood and Plant Proteins, and Fatty Acid ratio) score high by reaching recommended intake and three moderation components (Refined Grains, Sodium and Empty Calories) receive high scores for maintaining moderation. Intake evaluation is density based on food component intake per 1000 kcal or percent of calories and is scored proportionally when between minimum and maximum standards. Age and gender alter daily requirements; therefore, HEI uses the least restrictive recommendation to award the optimal score^(15)^. The maximum Total Score is 100 points with a higher score signifying closer compliance with 2010 DGA recommendations^(15)^ and a score of ≥80 is ideal^(3)^. While individual components can receive a score of 0, the scoring algorithm does not allow for a Total Score of 0. The algorithm for coding is available online^(26)^ and was implemented to convert the data generated by ASA24 to individual HEI-2010 component scores and total score with the simple HEI scoring algorithm method.

### Statistical analysis

After excluding subjects with questionable recalls, eighty-nine subjects were included in the analyses. Differences between continuous and categorical demographic data were assessed by two-sample *t*-tests and *χ*^2^ tests, respectively. Visual checks and the Kolmogorov–Smirnoff tests were conducted and indicated HEI-2010 scores were not normally distributed. Therefore, Wilcoxon sum-rank test was used to compare HEI-2010 scores by race (black *v.* white) and weight status (non-obese (BMI < 29⋅9) and obese (BMI ≥ 30)). The percentage of women in each subgroup who achieved maximum points for each HEI-2010 component was calculated. Statistical analysis was performed using SAS Version 9⋅4 (SAS Institute, Inc., Cary, NC). *P*-values < 0⋅05 were considered statistically significant. Comparisons for subcomponents for the HEI scores were not adjusted for multiple comparisons due to the exploratory nature of this substudy.

## Results

### Demographics

Among study participants, fifty women identified as black and thirty-nine as white (Table 1). Black women were significantly older than white women (mean ± standard error of the mean (sem): 42⋅5 ± 2⋅0 *v.* 36⋅1 ± 2⋅0; *P* = 0⋅03) and had a statistically higher mean BMI (33⋅9 ± 1⋅4 *v.* 27⋅6 ± 1⋅1; *P* < 0⋅001). The percentages of women who were non-obese and obese were statistically significantly different by race (*P* = 0⋅01). There was no statistically significant age difference by weight status. Education level and household income did not differ by race or weight status (Table 1).

**Table 1.** Participant demographics

	All		Black women		White women		*P*-value	Non-obese women		Obese women		*P*-value
	*n* 89		*n* 50		*n* 39			*n* 48		*n* 41		
	Mean	sem	Mean	sem	Mean	sem		Mean	sem	Mean	sem	
Age	39⋅7	1⋅4	42⋅5	2⋅0	36⋅1	2⋅0	0⋅03	38⋅6	2⋅1	40⋅9	1⋅9	0⋅43
Median (range)	40⋅0 (20–76)		41⋅5 (20–76)		33⋅0 (21–62)			38⋅5 (20–76)		41⋅0 (20–64)		
BMI	31⋅1	1⋅0	33⋅9	1⋅4	27⋅6	1⋅1	<0⋅01	24⋅7	0⋅4	38⋅7	1⋅3	<0⋅01
Median (range)	28⋅4 (17⋅7–61⋅0)		31⋅4 (20⋅4–61⋅0)		25⋅2 (17⋅7–51⋅4)			24⋅5 (17⋅7–29⋅3)		36⋅3 (30⋅1–61⋅0)		
Non-obese, *N* (%)	48 (53⋅9)		21 (42⋅0)		27 (69⋅2)		0⋅01					
Obese, *N* (%)	41 (46⋅1)		29 (58⋅0)		12 (30⋅1)							
Education^a^, *N* (%)												
≤High School	7 (8⋅0)		6 (12⋅0)		1 (2⋅6)		0⋅27	2 (4⋅2)		5 (12⋅5)		0⋅28
College	57 (64⋅8)		31 (62⋅0)		26 (68⋅4)			31 (64⋅6)		26 (65⋅0)		
Postgraduate	24 (27⋅3)		13 (26⋅0)		11 (29⋅0)			15 (31⋅3)		9 (22⋅5)		
Household Income, *N* (%)												
< 10 000	14 (15⋅7)		8 (16⋅0)		6 (15⋅4)		0⋅10	7 (14⋅6)		7 (17⋅1)		0⋅21
10 000–19 999	6 (6⋅7)		3 (6⋅0)		3 (7⋅7)			4 (8⋅3)		2 (4⋅9)		
20 000–29 999	7 (7⋅9)		4 (8⋅0)		3 (7⋅7)			2 (4⋅2)		5 (12⋅2)		
30 000–39 999	17 (19⋅1)		13 (26⋅0)		4 (10⋅3)			7 (14⋅6)		10 (24⋅4)		
40 000–49 999	17 (19⋅1)		12 (24⋅0)		5 (12⋅8)			8 (16⋅7)		9 (22⋅0)		
≥50 000	28 (31⋅5)		10 (20⋅0)		18 (46⋅2)			20 (41⋅7)		8 (19⋅5)		
Marital Status^b^, *N* (%)												
Single	35 (39⋅8)		19 (38⋅8)		16 (41⋅0)		0⋅10	19 (40⋅4)		16 (39⋅0)		0⋅96
Married	32 (36⋅4)		14 (28⋅6)		18 (46⋅2)			17 (36⋅2)		15 (36⋅6)		
Separated	3 (3⋅4)		3 (6⋅1)		0 (0⋅0)			1 (2⋅1)		2 (4⋅9)		
Divorced	14 (15⋅9)		9 (18⋅4)		5 (12⋅8)			8 (17⋅0)		6 (14⋅6)		
Widowed	4 (4⋅6)		4 (8⋅2)		0 (0⋅0)			2 (4⋅3)		2 (4⋅9)		

sem, standard error of the mean.

a Education level missing for one subject (white, obese).

b Marital status missing for on subject (black, non-obese).

### Calories and macronutrients of dietary recalls

The mean caloric intake for women in the combined sample was 1863⋅0 (sem 62⋅0 kcal). Women of both races and weight group reported similar caloric intake and macronutrient composition (Table 2). While not statistically significant, it is notable that obese women reported a lower intake of calories (60⋅1 kcal), protein (6⋅2 g) and carbohydrates (12⋅1 g).

**Table 2.** Calories, macronutrients, Healthy Eating Index scores by race and weight status

	Total		Black women		White women		*P*-value	Non-obese women		Obese women		*P*-value
Diet component calories and macronutrients*	*n* 89		*n* 50		*n* 39			*n* 48		*n* 41		
	Mean	sem	Mean	sem	Mean	sem		Mean	sem	Mean	sem	
Calories (kcal)	1863⋅0	62⋅0	1839⋅3	78⋅2	1893⋅4	101⋅0	0⋅38	1890⋅7	86⋅3	1830⋅6	89⋅9	0⋅63
Protein (g)	72⋅1	3⋅1	67⋅6	3⋅8	77⋅9	5⋅1	0⋅11	75⋅0	4⋅4	68⋅8	4⋅4	0⋅33
Total fat (g)	78⋅5	3⋅5	78⋅4	4⋅7	78⋅6	5⋅2	0⋅98	78⋅0	4⋅4	79⋅0	5⋅6	0⋅88
Carbohydrates (g)	221⋅9	8⋅7	221⋅6	10⋅8	222⋅4	14⋅4	0⋅96	227⋅5	12⋅8	215⋅4	11⋅6	0⋅49
HEI-2010 Component (Possible points)												
Total HEI-2010 Score (0–100)												
Median	51⋅9		50		52⋅8		0⋅34	56⋅9		46⋅1		0⋅04
IQR	39⋅1–63⋅4		39⋅1–60⋅9		37⋅4–70⋅2			39⋅9–70⋅5		37⋅3–57⋅8		
Total Vegetables (0–5)												
Median	4⋅1		3⋅7		5⋅0		0⋅10	4⋅1		4⋅2		0⋅77
IQR	2⋅7–5⋅0		2⋅6–5⋅0		2⋅7–5⋅0			2⋅3–5⋅0		2⋅9–5⋅0		
Greens and Beans (0–5)												
Median	0⋅4		0⋅2		0⋅9		0⋅45	0⋅4		0⋅5		0⋅73
IQR	0⋅0–5⋅0		0⋅0–5⋅0		0⋅0–5⋅0			0⋅0–5⋅0		0⋅0–5⋅0		
Total Fruit (0–5)												
Median	3⋅4		4⋅1		2⋅4		0⋅09	4⋅4		2⋅0		0⋅06
IQR	0⋅3–5⋅0		0⋅6–5⋅0		0⋅1–4⋅9			1⋅4–5⋅0		0⋅1–4⋅8		
Whole Fruit (0–5)												
Median	3⋅4		2⋅5		4⋅0		0⋅54	5⋅0		0⋅5		0⋅02
IQR	0⋅0–5⋅0		0⋅0–5⋅0		0⋅0–5⋅0			0⋅1–5⋅0		0⋅0–5⋅0		
Whole Grains (0–10)												
Median	1⋅0		1⋅2		0⋅6		0⋅77	1⋅1		0⋅1		0⋅23
IQR	0⋅0–4⋅9		0⋅0–4⋅7		0⋅0–5⋅8			0⋅0–5⋅3		0⋅0–2⋅3		
Dairy (0–10)												
Median	4⋅2		3⋅8		5⋅0		0⋅36	4⋅6		4⋅1		0⋅62
IQR	2⋅1–7⋅0		1⋅8–6⋅2		2⋅1–7⋅0			1⋅9–7⋅9		2⋅2–6⋅8		
Total Protein (0–5)												
Median	5⋅0		5⋅0		5⋅0		0⋅79	5⋅0		5⋅0		0⋅35
IQR	3⋅5–5⋅0		3⋅1–5⋅0		4⋅0–5⋅0			3⋅6–5⋅0		3⋅4–5⋅0		
Seafood and Plant Protein (0–5)												
Median	0⋅0		0⋅0		0⋅3		0⋅39	0⋅1		0⋅0		0⋅50
IQR	0⋅0–5⋅0		0⋅0–3⋅5		0⋅0–5⋅0			0⋅0–5⋅0		0⋅0–3⋅4		
Fatty Acid Ratio (0–10)												
Median	4⋅8		4⋅7		4⋅8		0⋅43	4⋅2		6⋅4		0⋅32
IQR	1⋅9–10⋅0		2⋅2–10⋅0		1⋅3–9⋅9			1⋅0–9⋅9		2⋅7–10⋅0		
Sodium^a^ (0–10)												
Median	2⋅4		2⋅4		1⋅6		0⋅68	3⋅1		1⋅0		0⋅20
IQR	0⋅0–5⋅7		0⋅0–6⋅0		0⋅0–5⋅6			0⋅0–6⋅7		0⋅0–4⋅2		
Refined Grains^a^ (0–10)												
Median	7⋅8		6⋅8		8⋅9		0⋅19	8⋅4		6⋅5		0⋅36
IQR	4⋅1–10⋅0		3⋅3–10⋅0		5⋅1–10⋅0			4⋅2–10⋅0		3⋅4–10⋅0		
Empty Calories^a^ (0–20)												
Median	13⋅0		12⋅6		13⋅0		0⋅51	15⋅0		10⋅8		0⋅05
IQR	8⋅5–17⋅0		8⋅4–16⋅4		8⋅9–17⋅4			10⋅2–17⋅9		8⋅4–15⋅7		

kcal, kilocalories; g, grams; sem, standard error of the mean; HEI, Healthy Eating Index; IQR, Interquartile Range.

* The daily caloric recommendation for sedentary women aged 26–50 is 1800 kcal^(27^^)^. The recommended grams of macronutrients at this calorie level are 86 g protein, 61 g fat and 234 g carbohydrate^(28^^)^.

a A higher score signifies moderation and closer compliance with DGA recommendations. Empty Calories are calories from solid fats, alcoholic beverages and added sugars.

### HEI scores

Table 2 presents the measures of central tendency for HEI-2010 scores for the total sample and stratified by race and weight status. The median Total HEI-2010 Score for the sample was 51⋅9 (IQR: 39⋅1–63⋅4). There were no statistically significant differences in Total HEI score or component scores by race. However, a few non-statistically significant results emerged. Black women demonstrated a higher Total Fruit component score (4⋅1 *v.* 2⋅4; *P* = 0⋅09) and white women received a higher Total Vegetable component score (5⋅0 *v.* 3⋅7; *P* = 0⋅10). Non-obese women had a higher Total HEI score than obese women (56⋅9 *v.* 46⋅1; *P* = 0⋅04). Non-obese women reported eating statistically significantly more foods in the Whole Fruits group (5⋅0 *v.* 0⋅5; *P* = 0⋅02). While not statistically significant, a higher intake of Total Fruits (4⋅4 *v.* 2⋅0; *P* = 0⋅06) and lower intake of Empty Calories (15⋅0 *v.* 10⋅8; *P* = 0⋅05; higher score indicates closer compliance with moderation recommendations) by non-obese women compared to obese women emerged.

### Percent meeting recommendations

Table 3 presents the percentages of women who earned the maximum points for each HEI-2010 component in the total sample and by race and weight status. Fewer black than white women achieved maximum points for Total Vegetables (26⋅0 % *v.* 51⋅3 %; *P* = 0⋅01), while significantly more black than white women achieved maximum points for Total Fruit (44⋅0 % *v.* 23⋅1 %; *P* = 0⋅01).

**Table 3.** Standards for maximum points and percent of women who achieved maximum points for HEI-2010 components

HEI-2010 Component	Standard for maximum score	Total	Black women	White women	*P*-value	Non-obese women	Obese women	*P*-value
		*n* 89	*n* 50	*n* 39		*n* 48	*n* 41	
		*n* (%)	←*n* (%)→			←*n* (%)→		
Total Vegetables	≥1⋅1 cup equiv/1000 kcal	33 (37⋅1)	13 (26⋅0)	20 (51⋅3)	0⋅01	18 (37⋅5)	15 (36⋅6)	0⋅93
Greens and Beans	≥0⋅2 cup equiv/1000 kcal	29 (32⋅6)	15 (30⋅0)	14 (35⋅9)	0⋅56	16 (33⋅3)	13 (31⋅7)	0⋅87
Total Fruit	≥0⋅8 cup equiv/1000 kcal	31 (34⋅8)	22 (44⋅0)	9 (23⋅1)	0⋅04	21 (43⋅8)	10 (24⋅4)	0⋅06
Whole Fruit	≥0⋅4 cup equiv/1000 kcal	40 (44⋅9)	22 (44⋅0)	18 (46⋅2)	0⋅84	27 (56⋅3)	13 (31⋅7)	0⋅02
Whole Grains	≥1⋅5 oz equiv/1000 kcal	6 (6⋅7)	2 (4⋅0)	4 (10⋅3)	0⋅24	4 (8⋅3)	2 (4⋅9)	0⋅52
Dairy	≥0⋅4 cup equiv/1000 kcal	13 (14⋅6)	7 (14⋅0)	6 (15⋅4)	0⋅85	7 (14⋅6)	6 (14⋅6)	0⋅99
Total Protein	≥2⋅5 oz equiv/1000 kcal	49 (55⋅1)	28 (56⋅0)	21 (53⋅9)	0⋅84	29 (60⋅4)	20 (48⋅8)	0⋅27
Seafood and Plant Protein	≥0⋅8 oz equiv/1000 kcal	25 (28⋅1)	11 (22⋅0)	14 (35⋅9)	0⋅15	19 (39⋅6)	6 (14⋅6)	<0⋅01
Fatty Acid Ratio	(PUFAs + MUFAs)/SFAs > 2⋅5	23 (25⋅8)	14 (28⋅0)	9 (23⋅1)	0⋅60	12 (25⋅0)	11 (26⋅8)	0⋅84
Sodium	≤1⋅1 g/1000 kcal	6 (6⋅7)	4 (8⋅0)	2 (5⋅1)	0⋅59	4 (8⋅3)	2 (4⋅9)	0⋅52
Refined Grains	≤1⋅8 oz equiv/1000 kcal	26 (29⋅2)	13 (26⋅0)	13 (33⋅3)	0⋅45	16 (33⋅3)	10 (24⋅4)	0⋅36
Empty Calories^a^	SoFAA ≤19 % of energy	12 (13⋅5)	7 (14⋅0)	5 (12⋅8)	0⋅87	8 (16⋅7)	4 (9⋅8)	0⋅34

HEI, Healthy Eating Index; kcal, kilocalories; cup equiv, cup equivalents; oz equiv, ounce equivalents; PUFAs, polyunsaturated fats; MUFAs, monounsaturated fats; g, grams; SFA, saturated fatty acids; SoFAA, solid fats, alcohol and added sugars.

a Calories from solid fats, alcoholic beverages and added sugars.

When non-obese and obese women were compared, more non-obese than obese women achieved maximum scores for Whole Fruit intake (56⋅3 % *v.* 31⋅7 %; *P* = 0⋅02) and protein from plant and seafood sources (39⋅6 *v.* 14⋅6; *P* < 0⋅01). Non-obese subjects consumed more Total Fruit (43⋅8 % *v.* 24⋅4 %; *P* = 0⋅06), although it did not reach statistical significance.

## Discussion

In this racially diverse sample of forty-eight non-obese and forty-one obese women, more dietary differences were observed by weight status than by race. Although no statistically significant differences in calories or macronutrients emerged by weight or race, differences in diet quality and food group intake were observed. Fewer black than white women met or exceeded the recommended intake of vegetables, while more black than white women met recommended intake for fruit. When comparing aggregate score by group, better overall diet quality, higher intake of fruits and fewer discretionary categories were observed in non-obese *v.* women with obesity. Additionally, when comparing the percentage of participants per group who achieved a maximum score, non-obese women reported more protein from plant and seafood sources than women with obesity. Combined, these data support that non-obese women report better adherence to the DGA compared to women with obesity. Regardless of race or weight status, the women in this sample from Birmingham, AL, USA, have suboptimal diet quality compared to the ideal HEI^(3)^.

The aggregate caloric intake of our sample was within range of the recommended daily caloric level for sedentary women ages 26–50^(27)^, although the recommended macronutrient targets at this calorie level were not met^(28)^. No statistically significant differences in caloric intake or diet quality by HEI-2010 were observed between races. Similar to our findings in women, HEI-2005 Total Scores calculated from 2003 to 2004 NHANES data by Hiza *et al.*^(11)^ did not differ between black and white adults although food group intake differed by race. White adults reported higher component scores for vegetables, whole grains and dairy, while black adults reported more ideal intakes for saturated fat and sodium^(11)^. We likewise observed that more white women reported the recommended amount of vegetables compared to black women. We also observed more black than white women consumed the recommended amount of total fruits. Similarly, a recent report by Thompson *et al.*^(29)^ observed non-Hispanic black men consumed more fruit yet fewer vegetables than their white male counterparts^(29)^. In our sample, racial differences in Total Score were not present likely because better total fruit intake in black women compensated for lower vegetable intake scores. This report in combination with Thompson *et al.* adds to the growing literature of minimal race difference in overall diet quality yet emphases potential race-tailored interventions aimed to improve diet quality.

In non-obese participants, we observed higher intake of whole fruits and HEI-2010 Total Score, better adherence to consuming protein from seafood and plant sources, and higher Total Fruits and Empty Calories component scores that did not reach statistical significance. However, even with superior diet quality, through increased intake of healthier food and better moderation of discretionary foods, there were no significant differences in total calorie or macronutrient intake between weight statuses. That is, women of both weight statuses reported similar caloric intake and macronutrient composition, but the health quality of foods composing the diet of non-obese participants was superior to the obese participants. We are not the first to observe a lower incidence of obesity in individuals with better diet quality. However, in contrast to our results, Jessri *et al.* observed decreased energy density in higher quality diets^(20)^.

It is notable that obese women reported a lower intake of calories, protein and carbohydrates. While statistical significance was not reached, this phenomenon is often indicative of underreporting intake. It is possible that the inverse association between HEI and weight is more robust than previously reported as obese individuals and those who desire weight loss are more likely to underreport intake, especially of energy-dense foods^(30)^, which could falsely improve HEI scores. Underreporting bias could underestimate the trend in poorer discretionary calorie moderation in obese participants in the present study. However, it is noteworthy few individuals in either group adhered to the moderation recommendation (16⋅7 % non-obese *v.* 9⋅8 % obese; *P*-value = 0⋅34) and moderation of alcohol, solid fats and added sugars should be a dietary goal regardless of weight status.

The HEI-2010 Total Score of 51⋅9 in this sample is lower than the first reported HEI score for United States citizens (63⋅8)^(17)^ and previously reported scores in the Southern United States. The mean HEI-2010 score was 57⋅8 from a multiple-site Southeastern study^(4)^, and in the Mississippi Delta, the 1999–2000 HEI version yielded a 60⋅1^(5)^ and the HEI-2005 was 54⋅5^(31)^. While we cannot neglect the possibility of regional dietary differences, supported by Hawaiian and Californian residents HEI Total Scores averaging in the low seventies^(21)^, an increasing age generally has a positive influence on diet quality^(11)^. In comparison to this study, this remains true in three samples of older US Southerners: a sample of older adults in rural North Carolina had 60⋅5 by the 1999–2000 HEI version^(32)^, another cohort of older adults in rural North Carolina scored 61⋅9 by HEI-2005^(3)^, and older adults in the Birmingham area previously scored an HEI-2005 of 57⋅9^(6)^. Unfortunately, the small sample size limited our ability to conduct a multivariable analysis of the HEI scores concerning age. Another potential explanation for why the Total Score for this sample may be lower than previous studies is the use of one 24-h recall in the present study, whereas those with multiple recalls^(6,32)^ or a food frequency questionnaire^(3,4,21)^ may more validly capture usual intake.

Some limitations of this study include the possibility of recall bias as well as purposeful underreporting and altering of the diet. An additional limitation of this study is the single 24-h recall and the choice to use the simple HEI scoring algorithm method, which limits the extrapolation to usual intake. However, this method allowed for comparison of individuals within each group (i.e. percentage of women in each subgroup who achieved maximum points for each HEI-2010 component). This sample includes women who are non-smokers and willing to be enrolled in a study requiring faecal sampling, which limits its representation of the rest of the female population in Birmingham, Alabama. Lastly, because this reports the findings of an ancillary study, the original sample size was not calculated to detect statistically significant differences in HEI-2010 scores by race or weight status. To better interpret our findings, we conducted *post hoc* power calculations and determined the *post hoc* power was only 46⋅7 % to detect differences in total HEI-2010 scores based on our sample size, summary statistics and alpha = 0⋅05. Thus, a focus on effect sizes may be more meaningful than indicators of statistical significance in informing the next steps. Even with these limitations, this study possesses several strengths. For example, we observed the expected outcome of better discretionary calorie intake in non-obese women even with the possible underreport of intake by obese women^(30)^. Additionally, our sample was relatively well balanced by race and weight status, and by using HEI, our results can be compared to other studies for regional and population differences.

Although only women were included in this study, these results are far-reaching. Long-term adherence to high-quality diets or improving one's diet is associated with lower accumulation of adipose tissue, which could improve some age-related diseases^(21)^. Diet quality can be improved by simple replacements (e.g. water instead of sugar-sweetened beverages and fruit instead of grain-based sweets)^(31)^. The practice and future research implications of the present results include not only racial and weight appropriate dietary interventions and long-term health improvements but also extend to the impact a woman may have on her environment. Given women currently retain the role of food gatekeepers in most households^(33)^, a woman's diet quality may transfer to others in her household, evidenced by the diet quality of young children is positively impacted by that of their mothers^(34)^. The effectiveness of a dietary intervention in women on partners and others in the household is less understood.

This study indicates that women in Birmingham, Alabama, USA, have suboptimal diet quality, which is consistent with other reports of poor dietary intake in the Southern United States. This study characterised behaviours that provide targets for tailored dietary interventions that are racially and weight appropriate, such as guidance for black women to increase vegetable intake, white women to consume more fruit, and obese women to target more whole fruits and diversify protein sources to include more seafood and plant-based protein.
